# Predicting radiological severity of pulmonary tuberculosis in children: an assessment of the WHO-criteria and novel prediction scores on an individual participant dataset

**DOI:** 10.64898/2026.07.07.26357441

**Published:** 2026-07-10

**Authors:** Akshita Gupta, Marieke M. van der Zalm, Minh Huyen Ton Nu Nguyet, Marc d’Elbée, Peter J. Dodd, Megan Palmer, Leyla Larsson, Alia Razid, Anneke C. Hesseling, Rory Dunbar, Norbert Heinrich, Heather J. Zar, Nyanda E. Ntinginya, Celso Khosa, Marriott Nliwasa, Valsan Philip Verghese, Maryline Bonnet, Eric Wobudeya, Bwendo Nduna, Raoul Moh, Juliet Mwanga-Amumpere, Ayeshatu Mustapha, Guillaume Breton, Jean-Voisin Taguebue, Laurance Borand, Pierre Goussard, H Simon Schaaf, Julie Morrison, Olivier Marcy, James A. Seddon, Chishala Chabala, Laura Olbrich, the RaPaed-TB Consortium, the Umoya Study Group, the TB Speed Consortium

## Abstract

**Background:**

The World Health Organization (WHO) recommends 4-month treatment for children with non-severe pulmonary tuberculosis, outlining eligibility criteria for settings with and without chest X-ray (CXR). We evaluated the diagnostic accuracy of the WHO eligibility criteria in settings without CXR (WHO-criteria) and developed clinical scores to support disease classification.

**Methods:**

Using data from an individual participant dataset (IPD; Decide TB) of children with confirmed/unconfirmed tuberculosis from four diagnostic studies (RaPaed-TB, Umoya, TB-Speed HIV, TB-Speed Decentralisation), we assessed the diagnostic accuracy of the WHO-criteria (with/without bacteriological testing) using expert CXR interpretation as a reference. We developed two multivariable logistic regression models with (Score 1) and without (Score 2) bacteriological testing, converted coefficients into integer scores with a threshold of >10 corresponding to a sensitivity ≥70%.

**Results:**

Of 2,383 children in the Decide TB IPD, 633 (26.6%) met the eligibility criteria for a 4-month regimen, of whom 116 (18.3%) had radiologically severe disease. With and without bacteriological testing, the WHO-criteria had sensitivities of 30.1% (95%CI: 20.3%-40.2%) and 21.7% (95%CI: 10.4%-34.5%), and specificities of 83.4% (95%CI: 80.2%-86.4%) and 81.9% (95%CI: 78.8%-84.9%), respectively. Score 1 and Score 2 had sensitivities of 41.1% (95%CI: 32.4%-49.5%) and 30.9% (95%CI: 22.6%-40.4%), and specificities of 77.3% (95%CI: 73.6%-80.8%) and 83.0% (95%CI: 79.5%-86.3%), respectively. Using WHO-criteria, 91/116 (78.4%) and 105/116 (90.5%) of children were at risk of undertreatment, compared to 68/116 (58.6%) and 80/116 (68.9%) when using developed scores.

**Conclusions:**

Developed scores demonstrated better sensitivity than WHO-criteria, however, performance remained suboptimal. Implementing shorter antituberculosis regimens without CXR remains challenging in children.

## Full Text Availability

The license terms selected by the author(s) for this preprint version do not permit archiving in PMC. The full text is available from the preprint server.

