# Predicting radiological severity of pulmonary tuberculosis in children: an assessment of the WHO-criteria and novel prediction scores on an individual participant dataset

**DOI:** 10.64898/2026.07.07.26357441

**Published:** 2026-07-20

**Authors:** Akshita Gupta, Marieke M. van der Zalm, Minh Huyen Ton Nu Nguyet, Marc d’Elbée, Peter J. Dodd, Megan Palmer, Leyla Larsson, Alia Razid, Anneke C. Hesseling, Rory Dunbar, Norbert Heinrich, Heather J. Zar, Nyanda E. Ntinginya, Celso Khosa, Marriott Nliwasa, Valsan Philip Verghese, Maryline Bonnet, Eric Wobudeya, Bwendo Nduna, Raoul Moh, Juliet Mwanga-Amumpere, Ayeshatu Mustapha, Guillaume Breton, Jean-Voisin Taguebue, Laurance Borand, Pierre Goussard, H Simon Schaaf, Julie Morrison, Olivier Marcy, James A. Seddon, Chishala Chabala, Laura Olbrich

**Affiliations:** 1 Institute of Infectious Diseases and Tropical Medicine, LMU University Hospital, LMU Munich; 2 German Centre for Infection Research (DZIF), Partner Site Munich, Munich, Germany; 3 Desmond Tutu TB Centre, Department of Paediatrics and Child Health, Faculty of Medicine and Health Sciences, Stellenbosch University, Cape Town, South Africa; 4 University of Bordeaux, National Institute for Health and Medical Research (Inserm) UMR 1219, Research Institute for Sustainable Development (IRD) EMR 271, Bordeaux, France; 5 Sheffield Centre for Health and Related Research, School of Medicine & Population Health, University of Sheffield, Sheffield, United Kingdom; 6 Clinical Research Department, Faculty of Infectious and Tropical Diseases, London School of Hygiene and Tropical Medicine, London, United Kingdom; 7 Fraunhofer Institute ITMP, Immunology, Infection and Pandemic Research, Munich, Germany; 8 Department of Paediatrics & Child Health, Red Cross War Memorial Children’s Hospital, MRC Unit on Child and Adolescent Health, University of Cape Town, South Africa; 9 NIMR - Mbeya Medical Research Centre, P. O. Box 2410, Mbeya, Tanzania; 10 Instituto Nacional de Saúde (INS), Maputo, Mozambique; 11 Faculty of Medicine, Eduardo Mondlane University, Maputo, Mozambique; 12 Kamuzu University of Health Sciences, Blantyre, Malawi; 13 Pediatric Infectious Diseases, Christian Medical College, Vellore, India; 14 TransVIMI, University of Montpellier, Institut de Recherche pour le Développement (IRD), Institut National de la Santé et Recherche Médicale (INSERM), Montpellier, France; 15 MU-JHU Care Ltd, Kampala, Uganda; 16 Arthur Davidson Children's Hospital, Ndola, Zambia; 17 Programme PAC-CI, Abidjan, Côte d’Ivoire; 18 Epicentre Mbarara Research Centre, Mbarara, Uganda; 19 Ola During Children Hospital, Fourah Bay Roadd, Freetown, Sierra Leone; 20 Solthis, Paris, France; 21 Mother and Child Center - Chantal Biya, Foundation, Yaoundé, Cameroon; 22 Epidemiology and Public Health Unit, Institut Pasteur du Cambodge, Phnom Penh, Cambodia; 23 Department of Paediatrics and Child Health, Stellenbosch University, Cape Town, South Africa; 24 Department of Infectious Disease, Imperial College London, London, United Kingdom; 25 University of Zambia, Department of Paediatrics and Child Health, School of Medicine, Lusaka, Zambia

**Keywords:** Treatment shortening, 4-month regimen, Treatment decision algorithm, paediatric, tuberculosis, pulmonary, treatment initiation, individual participant dataset, disease severity, prediction score, radiological severity

## Abstract

**Background:**

The World Health Organization (WHO) recommends 4-month treatment for children with non-severe pulmonary tuberculosis, outlining eligibility criteria for settings with and without chest X-ray (CXR). We evaluated the diagnostic accuracy of the WHO eligibility criteria in settings without CXR (WHO-criteria) and developed clinical scores to support disease classification.

**Methods:**

Using data from an individual participant dataset (IPD; Decide TB) of children with confirmed/unconfirmed tuberculosis from four diagnostic studies (RaPaed-TB, Umoya, TB-Speed HIV, TB-Speed Decentralisation), we assessed the diagnostic accuracy of the WHO-criteria (with/without bacteriological testing) using expert CXR interpretation as a reference. We developed two multivariable logistic regression models with (Score 1) and without (Score 2) bacteriological testing, converted coefficients into integer scores with a threshold of >10 corresponding to a sensitivity ≥70%.

**Results:**

Of 2,383 children in the Decide TB IPD, 633 (26.6%) met the eligibility criteria for a 4-month regimen, of whom 116 (18.3%) had radiologically severe disease. With and without bacteriological testing, the WHO-criteria had sensitivities of 30.1% (95%CI: 20.3%-40.2%) and 21.7% (95%CI: 10.4%-34.5%), and specificities of 83.4% (95%CI: 80.2%-86.4%) and 81.9% (95%CI: 78.8%-84.9%), respectively. Score 1 and Score 2 had sensitivities of 41.1% (95%CI: 32.4%-49.5%) and 30.9% (95%CI: 22.6%-40.4%), and specificities of 77.3% (95%CI: 73.6%-80.8%) and 83.0% (95%CI: 79.5%-86.3%), respectively. Using WHO-criteria, 91/116 (78.4%) and 105/116 (90.5%) of children were at risk of undertreatment, compared to 68/116 (58.6%) and 80/116 (68.9%) when using developed scores.

**Conclusions:**

Developed scores demonstrated better sensitivity than WHO-criteria, however, performance remained suboptimal. Implementing shorter antituberculosis regimens without CXR remains challenging in children.

## Introduction

The World Health Organization’s (WHO) Global Tuberculosis Report estimates that 1.2 million children and young adolescents (<15 years) developed tuberculosis in 2024, representing 11% of the global TB burden [1]. Two-thirds of children with pulmonary tuberculosis are estimated to have non-severe disease [2], most with smear-negative and non-severe forms of tuberculosis that could likely be treated for shorter durations [3]. Such treatment shortening may offer several advantages including reduced drug-induced adverse effects, improved adherence to treatment, reduction of costs of treatment to the family and the healthcare system [4–6].

In 2022, the SHINE trial demonstrated that a 4-month regimen of antituberculosis treatment was noninferior to a 6-month regimen in children with non-severe disease [3]. Based on these findings, the WHO issued operational guidelines outlining eligibility criteria for implementation of the 4-month regimen [4]. Classification of non-severe pulmonary tuberculosis under this guidance requires chest X-ray (CXR) evaluation to exclude radiologically severe disease [4]. Recognising that access to CXR may not be possible in many settings, the WHO provided additional recommendations for implementing the 4-month regimen where radiology was unavailable, (referred to as ‘WHO-criteria’) both with and without access to bacteriological testing (molecular testing and/or microscopy) (Supplemental table 1) [3,4]. In the absence of CXR, WHO recommends that children diagnosed with tuberculosis who present with mild symptoms not requiring hospitalization, with low bacillary burden (defined as smear negative or with semi-quantitative molecular testing results of negative, trace, very low or low) may be considered for the 4-month treatment regimen, provided these children are followed up monthly and respond well to treatment [4]. However, this recommendation is based on expert consensus with no evidence whether this approach ensures safe and effective treatment. This uncertainty poses challenges for implementation within national tuberculosis control programmes [6]. To facilitate implementation in low-resource settings, a data-driven approach predicting radiological severity of disease using available clinical indicators could complement existing WHO treatment decision algorithms (TDAs) in identifying children eligible for the 4-month regimen [4].

Using individual participant data (IPD) from children recruited to four existing well-characterised paediatric tuberculosis cohorts across eight countries [7], we sought to assess the WHO-criteria for starting a 4-month regimen with and without bacteriological testing. We developed two scores, with (Score 1) and without (Score 2) bacteriological testing to predict radiological severity of pulmonary tuberculosis in children.

## Methodology

### Study Design, population, and establishment of an IPD

A large individual participant dataset (IPD) was developed within the Decide TB project (EDCTP101103283) that included data from 2,383 children from four diagnostic studies (RaPaed-TB [8], Umoya [9], TB-Speed HIV [10,11], and TB-Speed Decentralisation [12]). In short, the IPD was prepared by extracting variables included in the TDAs (e.g. presence of symptoms, tuberculosis exposure, HIV status) and conducting subsequent standardisation before merging into a single dataset, to ensure that standard scales and definitions were used, including the creation of composite variables. Further details of the project [13], individual studies and variables in the IPD have been published [7] and are summarised in Supplemental table 2–3.

The WHO eligibility criteria are guidance for initiating the 4-month regimen in children with pulmonary tuberculosis as outlined in Box 5.3 from Module 5 of the Operational Handbook on Tuberculosis [4]. For this study, the WHO eligibility criteria in settings without CXR facilities is referred to as ‘WHO-criteria’ (i.e., described as Setting B and Setting C in Box 5.3 from Module 5 of the Operational Handbook; Supplemental table 1) [4]. Based on the WHO-criteria, we included children between 3 months and 10 years of age who were retrospectively classified as having either confirmed, unconfirmed or unlikely tuberculosis using National Institute of Health (NIH) 2015 clinical case definitions (Supplemental Table 4) [14], and were eligible for the 4-month regimen based on clinical presentation as outlined in the WHO-criteria (i.e., non-severe tuberculosis, without features precluding 4-month regimen use). Eligibility for the 4-month regimen under WHO-criteria is not synonymous with non-severe disease; only a subset of children with non-severe tuberculosis qualifies, as those with advanced HIV or severe acute malnutrition (SAM) remain ineligible irrespective of disease severity.

Thus, we excluded children with unlikely tuberculosis, extrapulmonary disease manifestations, without available Xpert MTB/RIF or Xpert MTB/RIF Ultra results, and children with severe clinical presentation as defined in the WHO operational handbook, being children living with HIV (CLHIV) with CD4 counts <100 cells/mm3 or unknown HIV status, SAM, respiratory distress, high fever (≥39°C), severe pallor, restlessness, irritability or lethargy.

### Radiographic Evaluations

As part of the original studies, all children had a CXR performed at baseline [7,15–17]. CXRs were retrospectively reviewed centrally by a single independent paediatric tuberculosis expert, to ensure uniformity across included studies. ‘Radiologically non-severe disease’ was defined using WHO definitions described in Setting A from in Box 5.3 from Module 5 of the Operational Handbook [4] as a) normal; or b) abnormal CXRs with any of the following features: intrathoracic lymph node tuberculosis without significant airway obstruction, or pulmonary tuberculosis confined to one lobe with no cavities and no miliary pattern, or uncomplicated pleural effusion (without pneumothorax or empyema). Abnormal CXRs with findings additional to those outlined (including multilobe presentations, airway obstruction, cavitary or miliary presentations, pneumothorax or empyema) were considered as ‘Radiologically severe disease’.

### Data analysis and reference standards

All data management and analysis were conducted using R version 4.4.2/ RStudio version 2024.12.1. Socio-demographic characteristics were summarised using proportions for categorical variables, and medians with interquartile ranges (IQR) or means with standard deviations (SD) for continuous variables. To assess the sensitivity and specificity of the WHO-criteria, we used CXR readouts from a single blinded expert as the reference standard for diagnostic accuracy estimation based on the definitions outlined above. Sensitivities and specificities were reported with 95% confidence intervals (CI) based on bootstrapping 1000 times. This analysis was repeated for predefined subgroups of interest (parent study, age, NIH 2015 case definition, Weight/BMI-for-age Z scores [WAZ], HIV status, healthcare level [Primary versus District versus Tertiary level], history of TB exposure).

### Prediction score development

We developed two multivariable logistic regression models with a random intercept to account for heterogeneity across parent studies: Model 1, including bacteriological testing, and Model 2, without bacteriological testing. The models included predictors from clinical features commonly considered during the evaluation of presumptive childhood pulmonary tuberculosis in primary healthcare settings and included in the WHO TDAs [4]. Details on the clinical predictors can be found in Supplemental table 3.

We performed internal validation for each model using bootstrap resampling 1000 times. The optimism estimate corresponded to the mean apparent-minus-test AUROC (area under the receiver-operating characteristic curve) difference across the bootstrap resamples. The optimism-corrected AUROC was calculated and reported as the apparent AUROC of the original fitted model minus the estimated optimism. We examined the ROC curves of both models to assess their discriminative ability. To facilitate the use of the model by clinicians, we translated the model into a scoring system, Score 1 (=Model 1) and Score 2 (=Model 2), where each predictor is assigned points on a scale of 10. We identified a range of different candidate logit thresholds (of predicted probabilities) corresponding to a sensitivity for classifying radiologically severe disease of at least 50%, and up to 90%. For each candidate threshold, we derived a scaling factor by subtracting the model intercept from the threshold and dividing by 10. Multiplying each predictor's β coefficient by this scaling factor yielded its integer point value (see Supplemental tables 5 and 6 for the final Score 1 and 2 development) [18]. We chose the final Score 1 and 2 after discussion based on: the balance between sensitivity and specificity for the threshold, the resulting integer values of each predictor after translation on a scale of 10, and final scores being practical for clinical use (see Supplemental methods).

Diagnostic accuracy was estimated for Score 1 and Score 2 compared to the reference standard as defined above, and mean sensitivities and specificities estimated through bootstrapping 1000 times were reported. Children at risk of undertreatment (false negatives) were defined as those children with radiologically severe disease who were incorrectly predicted as eligible for the 4-month regimen. Children at risk of overtreatment (false positives) were defined as those children with radiologically non-severe disease who were incorrectly predicted as eligible for the standard 6-month regimen.

## Results

### General population description

**Figure 1.**
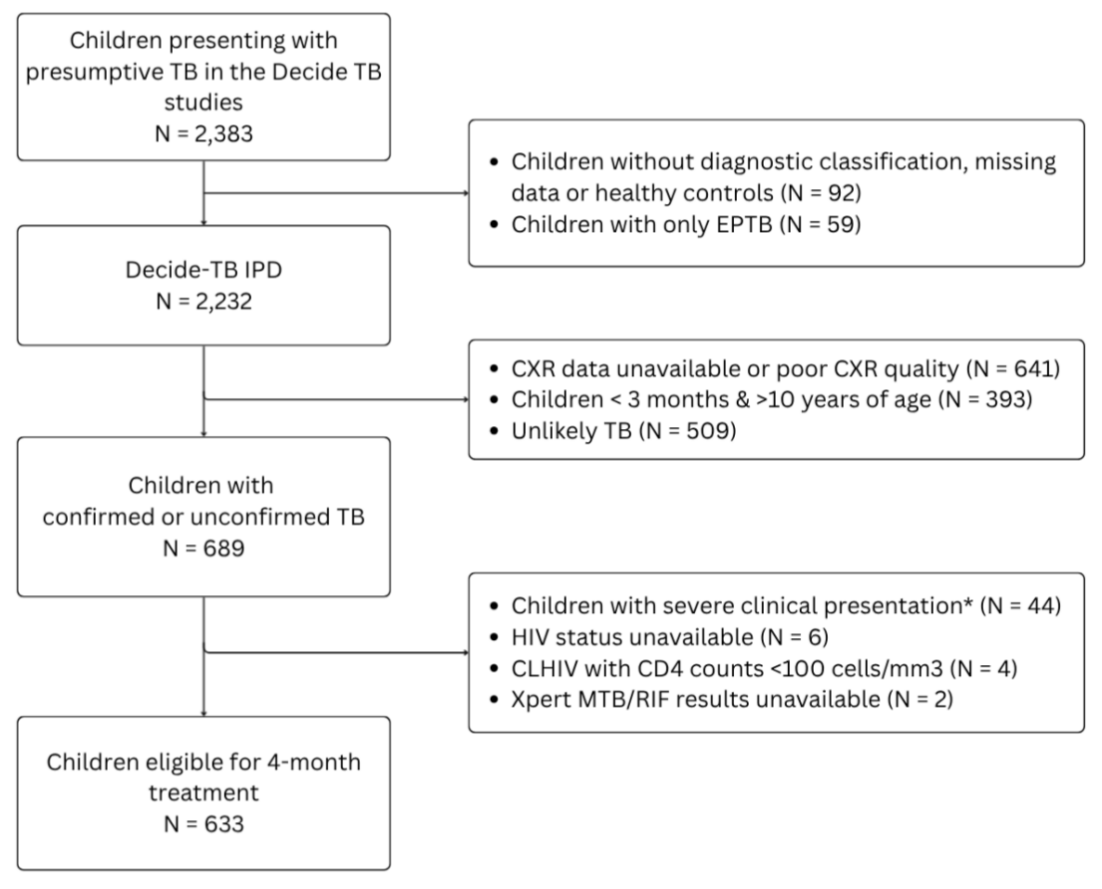
Development of the Decide TB individual participant dataset (IPD) for radiological severity prediction. Children were recruited from RaPaed-TB, Umoya, TB-Speed HIV, and TB-Speed Decentralization parent studies to build the Decide TB IPD. After applying inclusion and exclusion criteria, children who could be evaluated for 4-month regimen per the WHO eligibility criteria were included in the final analysis. *Eligibility for the 4-month regimen under the WHO criteria is not synonymous with non-severe disease as children may have non-severe TB and yet remain ineligible due to additional factors such as advanced HIV or severe acute malnutrition (SAM). Thus, we excluded children with severe clinical presentation as defined in the WHO operational handbook – severe acute malnutrition, respiratory distress, high fever (over 39°C), severe pallor, restlessness, irritability or lethargy [1]. IPD: Individual participant dataset; CXR: Chest X-ray; TB: Tuberculosis CLHIV: Children living with HIV. EPTB: extrapulmonary TB

Of 2,383 children included in the original studies (RaPaed-TB: n=975, Umoya: n=547, TB-Speed HIV: n=277, TB-Speed Decentralisation: n=584), 792 (33.2%) were excluded due to missing data or lack of diagnostic case classification, and a further 958 (40.2%) were excluded because their clinical presentation did not meet WHO-criteria for the 4-month regimen (Figure 1). Among the 633 children included in this analysis, the median age was 2.8 years (IQR: 1.3 – 5.2), 294 (46.4%) were female, and 118 (18.6%) were CLHIV. In total, 202 (31.9%) had confirmed tuberculosis and 431 (68.1%) unconfirmed tuberculosis. Most children had radiologically non-severe disease (517; 81.7%) and low bacterial burden (598; 94.5%) with semiquantitative Xpert MTB/RIF or Ultra results as negative or below medium (Table 1, Supplemental table 7).

### Evaluation of the WHO-criteria for classifying radiologically severe disease

The WHO-criteria with bacteriological testing had a sensitivity of 30.1% (95%CI: 20.3%-40.2%) and a specificity of 83.4% (95%CI: 80.2%-86.4%) for classifying radiologically severe disease. The WHO-criteria without bacteriological testing had a sensitivity of 21.7% (95%CI: 10.4%-34.6%) and a specificity of 81.9% (95%CI:78.8% - 84.9%). The WHO-criteria showed consistently low sensitivities across all subgroups, with and without bacteriological testing, with the lowest values observed in children aged 2 to 5 years (10.9% and 5.4% respectively) and those with WAZ > −2 to < −1 (14.4% and 2.8%). Irrespective of bacteriological testing, children without a history of TB exposure had higher sensitivity for radiologically severe disease than those with TB exposure (with bacteriology sensitivity of 27.3% vs 14.8% in tuberculosis- exposed subgroup; without bacteriology sensitivity of 16.0% vs 1.8% in tuberculosis-exposed subgroup; Supplemental Tables 8–9).

### Development and evaluation of prediction scores

**Figure 2.**
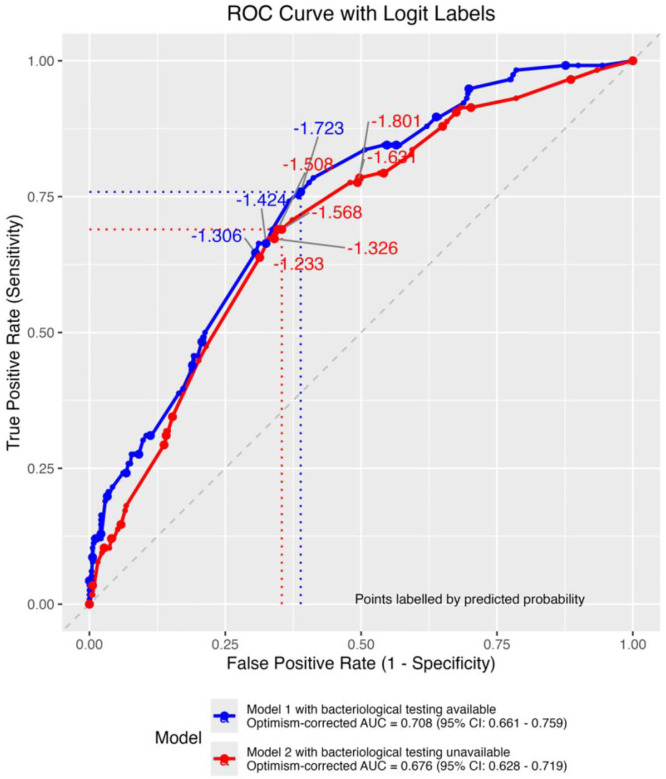
Receiver operating characteristic (ROC) curve for model 1 and model 2 in settings for logit selection and score development in dataset. (1) model 1 (blue) integrated all predictors including quantitative Xpert MTB/RIF or Ultra results. Optimism corrected AUC of model 1 was 0.708 (95%CI: 0.66–0.76), (2) Model 2 (red) excluded quantitative Xpert MTB/RIF or Ultra results. Optimism corrected AUC of model 2 was 0.676 (95%CI: 0.63–0.72). Points on the ROC curve indicate the candidate logit thresholds (predicted probabilities) of the model. A sensitivity of >70% was taken for selecting the cut off for the developed scores: −1.723 for model 1 (blue dotted line) and −1.568 for model 2 (red dotted line). AUC: Area under the curve

Model 1 was developed for settings with bacteriological testing and Model 2 for those without bacteriological testing available. Model 1 had an apparent AUROC of 0.732, with a mean optimism of 0.0234 (95%CI: −0.027 to 0.071), yielding an optimism-corrected AUROC of 0.708. Model 2 had an apparent AUROC of 0.699, with a mean optimism of 0.0226 (95%CI: −0.021 to 0.071), yielding an optimism-corrected AUROC of 0.676. We developed Score 1 based on Model 1 and Score 2 based on Model 2 (Figure 2; Supplemental Tables 5 and 6). The cut-off selection was driven by the balance between sensitivity and specificity for the threshold, the resulting integer values of each predictor after translation on a scale of 10, and final scores being practical for clinical use. For Score 1, we selected a cut-off on the ROC curve of Model 1 corresponding to a sensitivity of 75.9% (95%CI: 67.3–82.7) and a specificity of 61.5% (95%CI: 57.2–65.6); for Score 2, a cut-off on the ROC curve of Model 2 corresponding to a sensitivity of 68.9% (95%CI: 60.1–76.7) and a specificity of 64.8% (95%CI: 60.6–68.8) for the detection of radiologically severe pulmonary tuberculosis (Figure 2).

On diagnostic accuracy evaluation using the development dataset, Score 1 had a mean sensitivity of 41.1% (95%CI: 32.4%-49.5%) and mean specificity of 77.3% (95%CI: 73.6%-80.8%); while Score 2 had a mean sensitivity of 30.9% (95%CI: 22.6%-40.4%) and a mean specificity of 83.0% (95%CI: 79.5%-86.3%). The scores were evaluated among different subgroups of interest (Supplemental Tables 10–11). Across both scores, WAZ >−3 to <−2 was consistently associated with higher sensitivity (Score 1, 50.6% and Score 2, 42.3%) but the lowest specificity of any subgroup (Score 1, 65.2% and Score 2, 72.2%), and children without a history of tuberculosis exposure had higher sensitivity than those with known exposure in both scores (Score 1 sensitivity of 53.3% vs 28.0% in tuberculosis-exposed subgroup; Score 2 sensitivity of 40.6% vs 20.5% in tuberculosis-exposed subgroup). A schematic representation of how these scores could be incorporated into the WHO TDAs is shown in Supplemental Figure 1 and displays the score assigned to each clinical variable included in the models.

### Over- and undertreatment based on predicted radiological disease severity

**Figure 3.**
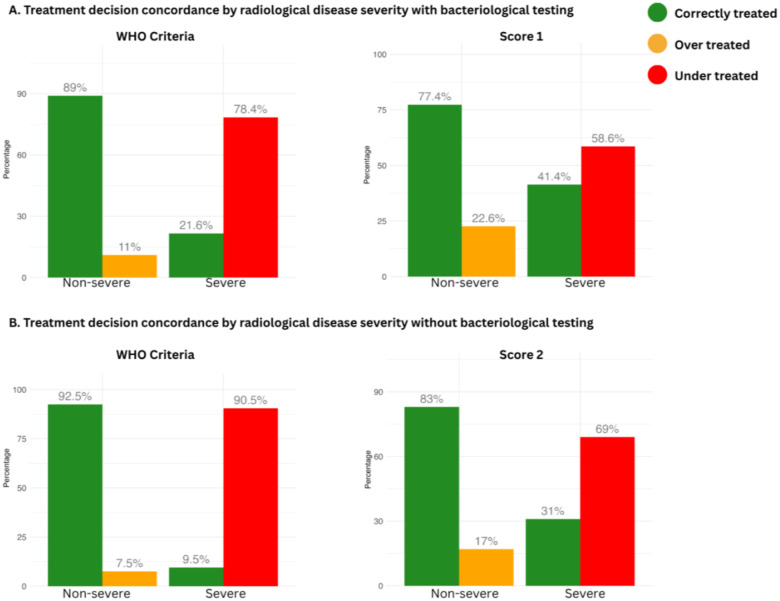
Treatment decision concordance in children predicted as severe or non-severe based on the WHO criteria and developed scores considering with and without bacteriological testing. Where Y-axis is the percentage of children and x-axis is the number of true radiologically non-severe (n = 517) and severe chest X-ray features (n = 116). (A) Classification of children based on the WHO-criteria and developed Score 1 with bacteriological testing (Setting B WHO Eligibility Criteria). (B) Classification of children based on the WHO-criteria and developed Score 2 without bacteriological testing (Setting C WHO Eligibility Criteria).

The WHO-criteria with bacteriological testing correctly identified 460/517 (88.9%) children with non-severe and 25/116 (21.6%) children with severe disease. Score 1 correctly identified 400/517 (77.4%) children with non-severe disease and 48/116 (41.4%) children as severe disease (Figure 3A, Supplemental Tables 8 and 10). Among children with severe disease, the WHO-criteria incorrectly identified 91/116 (78.4%) children as non-severe and Score 1 incorrectly identified 68/116 (58.6%) children as non-severe, placing them at risk of undertreatment.

The WHO-criteria without bacteriological testing correctly identified 478/517 (92.5%) children with non-severe and 11/116 (9.5%) of children with severe tuberculosis disease. Score 2 correctly identified 429/517 (82.9%) children as non-severe and 36/116 (31.0%) children as severe tuberculosis disease (Figure 3B, Supplemental Tables 9 and 11). The WHO-criteria led to 105/116 (90.5%) children incorrectly classified with severe disease, placing them at risk of undertreatment; while Score 2 led to 80/116 (68.9%) children incorrectly classified with severe disease, placing them at risk of undertreatment.

## Discussion

This is the first study to assess the performance of WHO eligibility criteria for the 4-month treatment regimen for settings where CXR is unavailable and to develop clinical prediction scores corresponding to radiological severity of pulmonary tuberculosis in children. The analysis leverages a large, well-characterised, and comprehensive IPD, covering a wide range of geographical regions with a high burden of tuberculosis. Our scores are intended for use alongside the WHO TDAs and to support identification of children eligible for the 4-month regimen when CXRs are not routinely available. While both scores improve radiological severity prediction compared with the current WHO-criteria, their overall performance remains suboptimal, resulting in a substantial risk of undertreatment among children with radiologically severe tuberculosis.

The WHO-criteria and Score 1 demonstrate better disease classification with bacteriological testing than without. However, given the paucibacillary nature of pulmonary tuberculosis in children [2,3] and the challenges in obtaining reliable respiratory samples [19], bacteriological confirmation is often not feasible. In such circumstances, disease severity would be evaluated primarily based on clinical symptoms, particularly in high-burden settings at lower (primary) levels of care [20]. The limitations of symptom-based severity classification are well-described, especially among children under 5 years in whom constitutional symptoms such as cough, fever, and weight loss are non-specific; overlapping with other common childhood illnesses and do not reliably reflect radiological tuberculosis disease severity [14,21–24].

Our analysis showed that using WHO-criteria or our scores carry a risk of misclassification leading to potential undertreatment of children with radiologically severe disease. The WHO-criteria address this concern by recommending a monthly follow up and allows for treatment to be extended to 6 months, if clinically indicated [4]. In the IPD overall, most children had unconfirmed tuberculosis and would require close follow-up during treatment. Furthermore, less than a third of children across the different studies had severe disease, in line with literature [2,3,5]. Taken together, these findings suggest that, although in practice the number of children at risk for undertreatment would be small, careful follow-up for response to therapy remains essential.

Based on our results, better access to affordable and interpretable radiology remains a critical need for the safe implementation of the 4-month regimen. In settings where radiological infrastructure exists but experienced CXR reviewers are lacking, computer-aided detection tools could potentially support severity assessment [25,26]. Such approaches could ultimately be used either to directly classify disease severity or to complement existing clinical decision-making tools such as the WHO TDAs or our scores [27]. Definitions of severe pulmonary tuberculosis in children rely largely on CXR features, and possible alternative approaches to disease classification methods that are less dependent on CXR may aid broader implementation of shorter regimens. Another consideration is the validity of radiological disease severity itself as a reference standard, as the SHINE trial enrolled children with only smear-negative, clinical and radiological non-severe disease. There is, however, no clear scientific evidence that the severe radiological features are indeed associated with more severe disease requiring longer TB treatment. Therefore, highlighting that there is no direct trial evidence that children with severe radiological features would fare worse on a shortened regimen. Taken together, these gaps point to a need for further research both on alternative approaches to severity classification and on the safety of shorter regimens across the full spectrum of disease severity.

Key limitations of this analysis are that the performance of our scores across subgroups was evaluated in the same dataset and external validation of the models in independent datasets was not performed. We used the retrospective NIH 2015 clinical case definitions for confirmed and unconfirmed tuberculosis as our inclusion criteria, as they provided a standardised, validated framework for tuberculosis classification enabling consistent groups across studies. Including all treated children would have introduced a heterogeneous population including potentially unlikely tuberculosis cases, risking contamination of the reference standard and biasing model performance. CXR interpretation forms part of the clinical case definitions which may introduce circularity when evaluating CXR-based predictors and potentially overstating utility. It is possible that the radiological features seen in some of the children classified as unconfirmed tuberculosis may have been attributable to alternative diagnoses, potentially impacting the accuracy of the models. In addition, the recruitment settings of the parent studies may limit generalisability, as most children were enrolled from district and tertiary level hospitals. This could potentially underrepresent primary level settings where a substantial burden of childhood tuberculosis exists, and radiological facilities or expert radiologists are often lacking. The CXR interpretation included in this analysis was performed by an experienced paediatric tuberculosis expert, which may not reflect the interpretation in routine clinical practice, where availability of experienced readers cannot always be assured. Lastly, this retrospective analysis could not assess the status of the child at the 4-month timepoint (reflecting end of treatment for non-severe disease) against the 6-month timepoint after starting treatment. Prospective studies comparing 4-month regimen outcomes are required to support implementation by national tuberculosis programs. Despite these limitations, our findings fill current knowledge gaps in implementing the 4-month regimen in children with pulmonary tuberculosis.

In conclusion, although our scores had better sensitivity than the current WHO-criteria, they are unable to reliably identify children with radiologically severe tuberculosis disease based on clinical features alone, with or without bacteriology. Implementing the 4-month regimen for children with pulmonary tuberculosis in the absence of adequate CXR infrastructure remains challenging and underscores the need for improved access to affordable and feasible CXR in resource-limited settings.

## Supplementary Material

1

## Figures and Tables

**Table 1.** Characteristics of children included in the individual participant dataset by radiological disease severity#

Characteristic	All Children in this IPD N = 633	Radiologically non-severe disease N = 517 (81.7%)	Radiologically severe disease N = 116 (18.3%)
**Study**			
RaPaed-TB (%)	272 (43.0%)	198 (38.3%)	74 (63.8%)
Umoya (%)	163 (25.8%)	146 (28.2%)	17 (14.7%)
TB-Speed HIV (%)	55 (8.7%)	47 (9.1%)	8 (6.9%)
TB-Speed Decentralization (%)	143 (22.6%)	126 (24.4%)	17 (14.7%)
**Age (years) [median (IQR)]**	2.81 (1.26, 5.21)	2.82 (1.35, 5.29)	2.67 (0.97, 5.07)
**Age (categories)**			
3 months to <2 years (%)	249 (39.3%)	198 (38.3%)	51 (44.0%)
2 to <5 years (%)	214 (33.8%)	178 (34.4%)	36 (31.0%)
5 to <10 years (%)	170 (26.9%)	141 (27.3%)	29 (25.0%)
**Sex**			
Female (%)	294 (46.4%)	239 (46.2%)	55 (47.4%)
Male (%)	339 (53.6%)	278 (53.8%)	61 (52.6%)
**Weight/BMI for Age (Z score)**			
> −1 (%)	341 (53.9%)	283 (54.7%)	58 (50.0%)
> −2 to < −1 (%)	187 (29.5%)	153 (29.6%)	34 (29.3%)
> −3 to < −2 (%)	105 (16.6%)	81 (15.7%)	24 (20.7%)
**CLHIV (%)**	118 (18.6%)	91 (17.6%)	27 (23.3%)
**NIH 2015 case definition**			
Confirmed tuberculosis (%)	202 (31.9%)	149 (28.8%)	53 (45.7%)
Unconfirmed tuberculosis (%)	431 (68.1%)	368 (71.2%)	63 (54.3%)
**Facility type**			
Primary Healthcare (%)	4 (0.6%)	4 (0.8%)	0 (0.0%)
District Healthcare (%)	139 (22%)	122 (23.6%)	17 (14.7%)
Tertiary Healthcare (%)	490 (77.4%)	391 (75.6%)	99 (85.3%)
**Symptoms**			
Cough ≥ 2 weeks (%)	427 (67.5%)	351 (67.9%)	76 (65.5%)
Fever ≥ 2 weeks (%)	256 (40.4%)	211 (40.8%)	45 (38.8%)
Weight loss (%)	358 (56.6%)	282 (54.5%)	76 (65.5%)
Lethargy (%)	276 (43.6%)	206 (39.8%)	70 (60.3%)
Haemoptysis (%)	14 (2.2%)	7 (1.4%)	7 (6.0%)
Night sweats (%)	220 (34.8%)	185 (35.8%)	35 (30.2%)
Enlarged lymph nodes (%)	110 (17.4%)	85 (16.4%)	25 (21.6%)
Tachycardia (%)	53 (8.4%)	35 (6.8%)	18 (15.5%)
Tachypnoea (%)	103 (16.3%)	74 (14.3%)	29 (25.0%)
**TB-related findings**			
TB Exposure (%)	360 (56.9%)	306 (59.2%)	54 (46.6%)
Anti-tuberculosis treatment initiated (%)	592 (93.5%)	482 (93.2%)	110 (94.8%)
**Bacteriological Testing**			
**Xpert MTB/RIF or Ultra**			
*Mtb* Negative (%)	494 (78.0%)	417 (80.7%)	77 (66.4%)
*Mtb* Positive, including trace (%)	139 (22.0%)	100 (19.3%)	39 (33.6%)
**Ziehl-Neelsen smear**			
Negative (%)	217 (94.3%)	187 (96.9%)	30 (81.1%)
Positive (%)	13 (5.7%)	6 (3.1%)	7 (18.9%)
Not performed (%)	403	324	79
**WHO eligibility criteria groups**			
Xpert MTB/RIF or Ultra results: Negative, Trace, Low, very low OR smear negative (%)	598 (94.5%)	499 (96.5%)	99 (85.3%)
Xpert MTB/RIF or Ultra results: Medium, high, very high OR smear positive (%)	35 (5.5%)	18 (3.5%)	17 (14.7%)

# Radiologically non-severe disease was defined using WHO definitions in Module 5 of the Operational Handbook as a) normal or b) abnormal CXRs with any of the following: intrathoracic lymph node tuberculosis without significant airway obstruction, or pulmonary tuberculosis confined to one lobe with no cavities and no miliary pattern, or uncomplicated pleural effusion (without pneumothorax or empyema). Abnormal CXRs with findings additional to those outlined (including but not limited to, multilobe presentations, airway obstruction, cavitary presentations, miliary presentations, pneumothorax or empyema etc.) were considered as radiologically severe disease.

*Mtb*: *Mycobacterium tuberculosis* TB: Tuberculosis; IPD: Individual participant dataset; NIH 2015 diagnosis: National Institute of Health (NIH 2015) clinical case definitions. BMI: Body Mass Index, CHLIV : Children living with HIV

## Data Availability

The IPD will be made available upon reasonable request with investigator support and a signed data access agreement. Submit a request to Alberto Beyersdorff, data manager at LMU Munich (Alberto.Beyersdorff@med.uni-muenchen.de). The code for the analysis is available at https://github.com/4Gupt4/Decide-TB.
