# Supplementary material for: Predicting radiological severity of pulmonary tuberculosis in children: an assessment of the WHO-criteria and novel prediction scores on an individual participant dataset
Source: medRxiv. 2026 Jul 20:2026.07.07.26357441. Originally published 2026 Jul 10. Preprint. [Version 2] doi: 10.64898/2026.07.07.26357441 (PMC13370569; doi:10.64898/2026.07.07.26357441)
Supplement: Supplementary file 1 [file NIHPP2026.07.07.26357441V2-supplement-1.pdf]

medRxiv preprint doi: https://doi.org/10.64898/2026.07.07.26357441; this version posted July 20, 2026. The copyright holder for this preprint (which was not certified by peer review) is the author/funder, who has granted medRxiv a license to display the preprint in perpetuity.
It is made available under a CC-BY 4.0 International license .

# Predicting radiological severity of pulmonary tuberculosis in children: an assessment of WHO criteria and novel prediction scores on an individual participant dataset

## Table of Contents

*Supplemental Methods* ........ 2

Study Design, population, and establishment of an IPD ........ 2

Prediction score development ........ 2

*Supplemental Table 1. Box 5.3 Eligibility criteria for the 4-month regimen (2HRZ(E)/2HR) in children and adolescents aged between 3 months and 16 years, in various settings.* ........ *4*

*Supplemental Table 2: Study characteristics of individual studies included in the IPD [1,4]* ........ *5*

*Supplemental Table 3: Study Variables [1]* ........ *8*

*Supplemental Table 4. Outcome definitions used in study, based on NIH case definitions [5].* ........ *14*

*Supplemental Table 5. Score 1 development for Model 1, with bacteriological testing.* ........ *15*

*Supplemental Table 6. Score 2 development for Model 2, without bacteriological testing.* ........ *16*

*Supplemental Table 7. Characteristics of children included in the individual patient dataset by parent study.* ........ *17*

*Supplemental Table 8. Diagnostic accuracy of the WHO-criteria where bacteriological testing available and in subgroups against radiological severity as the reference standard.* ........ *19*

*Supplemental Table 9. Diagnostic accuracy of the WHO-criteria where bacteriological testing unavailable and in subgroups against radiological severity as the reference standard.* ........ *21*

*Supplemental Figure 1. Scoring system schematic for possible use in practice for different settings alongside the WHO Treatment Decision Algorithms in the absence of chest X-ray.* ........ *23*

*Supplemental Table 10. Diagnostic accuracy of the developed score with bacteriological testing for predicting CXR severity in different subgroups.* ........ *24*

*Supplemental Table 11. Diagnostic accuracy of the developed scores without bacteriological testing for predicting CXR severity in different subgroups.* ........ *26*

*References* ........ *28*

medRxiv preprint doi: https://doi.org/10.64898/2026.07.07.26357441; this version posted July 20, 2026. The copyright holder for this preprint (which was not certified by peer review) is the author/funder, who has granted medRxiv a license to display the preprint in perpetuity.
It is made available under a CC-BY 4.0 International license .

## Supplemental Methods

### Study Design, population, and establishment of an IPD

In the TB-Speed HIV cohort (n=72; 10.6% of the entire IPD), data regarding night sweats was not collected. A simple imputation strategy based on a decision tree model was employed. The children recruited to the TB-Speed HIV, RaPaed-TB, and Umoya studies were recruited from tertiary level facilities, whereas children recruited to the TB-Speed Decentralization study were recruited from district and primary health care centres. A dataset including only the children living with HIV (from studies which are similar in inclusion criteria to TB-Speed HIV) from RaPaed-TB (n=148), Umoya (n=42), and TB-Speed HIV (n=204) was generated; and with the simputation package in R (version 0.2.8), we used a classification decision tree approach to predict night sweats status based on clinically relevant variables (age, history of tuberculosis exposure, severe acute malnutrition, cough ≥2 weeks, fever ≥2 weeks, weight loss, cavitations on chest X-ray, and Xpert result [Mtb positive or Mtb negative]) using the function impute_cart() [1]. The imputed dataset was then used for subsequent analyses.

### Prediction score development

The prediction model was developed for children under 10 years, reflecting the target age range of the WHO treatment decision algorithms (TDAs), even though WHO eligibility criteria for the 4-month treatment regimen extend to children under 16 years.

Final models were derived using backward stepwise selection based on the Akaike Information Criterion (AIC). This corresponds to retaining predictors with likelihood-ratio test statistics more than twice the degrees of freedom, approximately equivalent to using p-value thresholds of 0.157 for 1 degree of freedom and 0.135 for 2 degrees of freedom, as recommended for clinical prediction modelling to reduce overfitting and optimism [2]. We included 11 identical categorical clinical predictors: age (3 months to 2 years; 2 years to 5 years; 5 years to 10 years), sex (female; male), Weight/BMI for Age Z-Score (>-3 to <-2; >-2 to <-1; >-1), cough lasting ≥2 weeks (Yes; No), fever lasting ≥2 weeks (Yes; No), history of tuberculosis (TB) exposure (Yes; No), HIV status (Positive; Negative), night sweats (Yes; No), palpable lymph nodes (Yes; No), tachycardia based on age (Yes; No), tachypnoea based on age (Yes; No) (Supplemental table 3). Model 1 included all 11 predictors and additionally bacteriological testing including, smear microscopy (Positive; Negative) or semiquantitative Xpert MTB/RIF or Ultra (Positive: medium, high or very high; Negative: negative, trace, very low, low) results. Model 2 included only the 11 clinical predictors.

For translation into a score, we selected a target sensitivity of >70% to balance missed severe cases against the impact of excessive false positives in low-prevalence settings. Higher sensitivity targets (e.g. ≥85%) were considered but were not selected because they lie at the extreme upper tail of the ROC curve, where small changes in predicted risk result in large shifts in specificity and classification leading to instability and a marked loss of performance. Therefore, a moderate-sensitivity operating point was selected to support practical triage decisions while maintaining robustness after translation from the regression model to the integer score.

## Supplemental Table 1. Box 5.3 Eligibility criteria for the 4-month regimen (2HRZ(E)/2HR) in children and adolescents aged between 3 months and 16 years, in various settings.

Adapted from the WHO operational handbook [3]. For this study, 'WHO criteria' refers to Setting B and Setting C.

| | **Setting** | **Criteria** |
|---|---|---|
| Eligibility criteria for the 4-month regimen (2HRZ(E)/2HR) in children and adolescents aged between 3 months and 16 years, in various settings | A<br>In children and adolescents who have undergone Bacteriological testing and CXR, a 4-month treatment regimen should be started in children and adolescents meeting **all of the following three criteria** | (1) CXR findings consistent with non-severe TB. CXR should ideally be done at baseline:<br>a. intrathoracic lymph node TB without significant airway obstruction; OR<br>b. PTB confined to one lobe with no cavities and no miliary pattern; OR<br>c. uncomplicated pleural effusion (without pneumothorax or empyema)<br>(2) Xpert MTB/RIF or Ultra is negative, trace, very low or low, (Xpert MTB/RIF <Medium) or smear-negative (if Xpert MTB/RIF or Ultra not available)<br>(3) the child has mild symptoms that do not require hospitalization. |
| | B<br>In settings without CXR, children and adolescents who have undergone Bacteriological testing, a 4-month treatment regimen should be started in children and adolescents meeting **the following criteria** | (1) TB that is negative, trace, very low or low using Xpert MTB/RIF or Ultra (Xpert MTB/RIF <Medium), or smear-negative (if Xpert MTB/RIF or Ultra not available); AND<br>(2) the child or adolescent has mild symptoms that do not require hospitalization.<br>OR<br>(1) Isolated extra-thoracic (peripheral) lymph node TB, without confirmed or suspected involvement of other extrapulmonary sites of disease; AND<br>(2) the child or adolescent has mild symptoms that do not require hospitalization. |
| | C<br>In settings without CXR and Bacteriological testing, a 4-month treatment regimen should be started in children and adolescents meeting **either of the following criteria** | (1) Isolated extra-thoracic (peripheral) lymph node TB, without confirmed or suspected involvement of other extrapulmonary sites of disease; AND<br>(2) the child has mild symptoms that do not require hospitalization.<br>OR<br>(1) the child has a clinical diagnosis of pulmonary TB; AND<br>(2) the child has mild symptoms that do not require hospitalization. |

Mild symptoms that do not require hospitalization include the absence of danger or high-priority signs, absence of asymmetrical or persistent wheezing, and none of the following: SAM, respiratory distress, high fever (>39°C), severe pallor, restlessness, irritability.

## Supplemental Table 2: Study characteristics of individual studies included in the IPD [1,4]

| | RaPaed-TB | Umoya | TB-Speed decentralization | TB-Speed HIV |
|---|---|---|---|---|
| Aim | To evaluate several novel index tests and diagnostic approaches | To i) build a clinical, radiological, and biological sample repository to evaluate new emerging diagnostic tools and biomarkers and ii)) investigate the short and long-term impact of pulmonary TB and other respiratory illnesses on lung health and QoL | To assess the impact of implementing decentralized diagnostic approaches on childhood TB case detection at district hospital (DH) and primary health centres (PHCs) and to compare decentralization strategies | To externally validate the PAANTHER score in CLHIV with presumptive TB |
| Design | Prospective single-gate diagnostic accuracy study | Prospective observational cohort study | Cross-sectional operational research study with nested prospective cohort | Prospective diagnostic cohort for external validation of TDA |
| Population | Children < 15 years with presumptive TB | Children < 13 years with presumptive TB | Children <15 years with presumptive TB | CLHIV <15 years with presumptive TB |
| Duration | Participant recruitment was conducted between January 21, 2019, and July 1, 2021 | Participant recruitment was conducted from November 2017 to May 2023 | During study intervention period (March 7, 2020 to September 30, 2021), we decentralized the comprehensive childhood TB diagnosis package and documented TB diagnosis. The exact start and end dates varied across countries. | Participant recruitment was conducted between October 2, 2019 and December 31, 2021 |

| | | | | |
|---|---|---|---|---|
| Setting | South Africa, Mozambique, Malawi, India, Tanzania | South Africa | Cambodia, Cameroon, Côte d'Ivoire, Mozambique, Sierra Leone, Uganda | Côte d'Ivoire, Mozambique, Uganda, Zambia |
| Number screened | 5,313 | 1,330 | 584 | 713 |
| Number recruited | 975 | Up to 600 children with presumptive pulmonary TB and 100 healthy controls | 3,104 / 584 | 277 |
| Inclusion criteria | Consent and assent (if applicable), confirmation of TB disease and/or signs and symptoms suggestive of TB | Consent and assent (if applicable), <13 years, > 2.5 kg, presumptive pulmonary TB | For assessing decentralized diagnostic: Sick children seeking care at OPD of DH or PHC, age <15 years<br><br>For comparing decentralization strategies: Age <15 years, Presumptive TB defined as children presenting ≥1 systematic screening criterion[1], Presumptive TB identified by the site clinician irrespective of the above criteria, especially presumed extra-pulmonary TB cases, Informed consent signed, Child's assent obtained in those aged >7 years | Children aged 1 month to 14 years, Documented HIV-infection, Presumptive TB based on at least one criteria4, History of contact with a TB case and any of the symptoms[2] with a shorter duration (< 2 weeks), Informed consent signed by parent/guardian |
| Exclusion criteria | Critical condition posing an undue risk to the child, body weight < 2 Kg, age ≥ 15 years, currently receiving anti-TB drugs | Extra-thoracic TB only, TB treatment for >2 days in previous 2 weeks, Alternative diagnosis at baseline, Severe illness resulting in unstable clinical condition, Contra- | For assessing decentralized diagnostic: None<br><br>For comparing decentralization strategies: Children who | Ongoing TB treatment or history of intake of anti-TB drugs in the last 3 months (isoniazid alone or rifampin/isoniazid for preventive therapy is not an exclusion criteria) |

| | | | | |
|---|---|---|---|---|
| | | indication to sampling procedures, Discharge before baseline sampling completed, Unstable social circumstances, Residence in remote areas | have received TB treatment in the past 6 months | |
| Chest X-ray review for reference variable | CXRs were reviewed by single expert | CXRs were reviewed by single expert | CXRs were reviewed by single expert | CXRs were reviewed by single expert |

[1] Cough with a duration of >2 weeks, Fever with a duration of >2 weeks, Documented weight loss, History of TB contact with any duration of cough

[2] Persistent cough for more than 2 weeks, persistent fever for more than 2 weeks, recent failure to thrive (documented clear deviation from a previous growth trajectory in the last 3 months or Z score weight/age < 2), failure of broad-spectrum antibiotics for treatment of pneumonia, suggestive CXR features

Abbreviations: TB: Tuberculosis, QoL: Quality of Life, OPD: outpatient department, PHC: primary health care level, DH: district health level, CLHIV; Children Living with HIV, TDA: Treatment Decision Algorithm.

## Supplemental Table 3: Study Variables [1]

| Variable | Definitions | RaPaed | UMOYA | TB-Speed HIV | TB-Speed Decentralisation |
|---|---|---|---|---|---|
| Age groups | 3 months to <2 years; 2 years to <5 years; 5 years to 10 years | Date of birth or age (years) | Date of birth or age (years) | Date of birth or age (years) | Date of birth or age (years) |
| Cough ≥ 2 weeks | Persistent, unremitting cough for 2 weeks or more | Presence of cough at enrolment (binary) and cough duration | Presence of cough at enrolment and classification into acute (incl. number of days) or chronic (>2 weeks) | Presence of cough in the 4 weeks preceding enrolment (binary) and duration | Presence of cough > 2 weeks at enrolment (binary) |
| Weight/BMI for Age (Z score) | Anthropometric Z-scores for weight-for-age and BMI-for-age calculated from measured weight and height using WHO approved methods including R packages: Anthro for children <5 years; Anthroplus for children ≥5 years | Weight-for-age Z-score for children <5 years and BMI-for-age Z-score for children aged 5–10 years (continuous) | Weight-for-age Z-score for children <5 years and BMI-for-age Z-score for children aged 5–10 years (continuous) | Weight-for-age Z-score for children <5 years and BMI-for-age Z-score for children aged 5–10 years (continuous) | Weight-for-age Z-score for children <5 years and BMI-for-age Z-score for children aged 5–10 years (continuous) |
| Fever ≥ 2 weeks | Persistent fever for 2 weeks or more (the score in the algorithm is based on the duration of fever as per the history rather than the | Presence of fever at enrolment (binary) and duration of fever | Presence of fever at enrolment (binary) and duration of fever | Presence of fever in the 4 weeks preceding enrolment (binary) and duration | Presence of fever > 2 weeks at enrolment (binary) |

| | | | | | |
|---|---|---|---|---|---|
| | actual temperature on examination) | | | | |
| Night sweats | Excessive night-time sweating that soaks the bed or clothes | Presence of night sweats at enrolment (binary) | Presence of night sweats at enrolment (binary) | **Missing/not collected** | Presence of night sweats at enrolment (binary) only in children ≥ 5 years |
| Swollen lymph nodes | Non-painful, enlarged cervical, submandibular or axillary lymph nodes | Presence of lymph node at enrolment (binary) which are not characterised as painful, are bigger than 1cm, and are cervical, axillar, or submandibular | Presence of lymphadenopathy at enrolment (binary) which are bigger than 1.5cm, and are cervical, axillar, or submandibular | Presence of cervical or supra-clavicular Adenopathy (no, single, multiple) | Presence of adenopathy at enrolment (binary) with specified location (supra-clavicular or cervical), number, and size of biggest node (1-3 cm or >3 cm) |
| Tachycardia | Children aged under 2 months: heart rate over 160 beats/minute<br><br>Children aged 2–12 months: heart rate over 150 beats/minute<br><br>Children aged 12 months to 5 years: heart rate over 140 beats/minute | Children aged under 2 months: heart rate over 160 beats/minute<br><br>Children aged 2–12 months: heart rate over 150 beats/minute<br><br>Children aged 12 months to 5 years: | Children aged under 2 months: heart rate over 160 beats/minute<br><br>Children aged 2–12 months: heart rate over 150 beats/minute<br><br>Children aged 12 months to 5 years: | Children aged under 1 year: heart rate over 160 beats/minute<br><br>Children aged 1–2 years: heart rate over 150 beats/minute<br><br>Children aged 2-5 years: heart rate over 140 beats/minute | Children aged under 2 months: heart rate over 160 beats/minute<br><br>Children aged 2–12 months: heart rate over 150 beats/minute<br><br>Children aged 12 months to 5 years: |

| | | | | | |
|---|---|---|---|---|---|
| | Children aged over 5 years: heart rate over 120 beats/minute | heart rate over 140 beats/minute<br><br>Children aged over 5 years: heart rate over 120 beats/minute | heart rate over 140 beats/minute<br><br>Children aged over 5 years: heart rate over 120 beats/minute | Children aged over 5 years: heart rate over 120 beats/minute | heart rate over 140 beats/minute<br><br>Children aged over 5 years: heart rate over 120 beats/minute |
| Tachypnoea | Children aged under 2 months: respiratory rate over 60/minute<br><br>Children aged 2–12 months: respiratory rate over 50/minute<br><br>Children aged 12 months to 5 years: respiratory rate over 40/minute<br><br>Children aged over 5 years: respiratory rate over 30/minute | Children aged under 2 months: respiratory rate over 60/minute<br><br>Children aged 2–12 months: respiratory rate over 50/minute<br><br>Children aged 12 months to 5 years: respiratory rate over 40/minute<br><br>Children aged over 5 years: respiratory rate over 30/minute | Children aged under 2 months: respiratory rate over 60/minute<br><br>Children aged 2–12 months: respiratory rate over 50/minute<br><br>Children aged 12 months to 5 years: respiratory rate over 40/minute<br><br>Children aged over 5 years: respiratory rate over 30/minute | Not collected but respiratory rate and age available | Children aged under 2 months: respiratory rate over 60/minute<br><br>Children aged 2–12 months: respiratory rate over 50/minute<br><br>Children aged 12 months to 5 years: respiratory rate over 40/minute<br><br>Children aged over 5 years: respiratory rate over 30/minute |
| HIV status | | Overall HIV status generated based on reported status and test results. Overall HIV test results based on the combination of the first test with the | Overall HIV status generated based on reported status and test results | Entire cohort is living with HIV based on inclusion criteria for the study | HIV status following testing and in children < 18 months, an additional PCR was done |

| | | | | | |
|---|---|---|---|---|---|
| | | second and third confirmatory test | | | |
| SAM | | Based on weight for length Z-score and BMI-for-age Z-score < -3 (weight for length used in those under 5) | If weight for length <-3 Z-score or bilateral pitting oedema for those under 5 months of age<br>If weight for length <-3 Z-score or MUAC<11.5cm (with or without oedema for those over 5 months) | BMI-for-age Z-score < -3 (weight for height in those under 5); MUAC; presence of oedema | Weight for height Z-score < -3 if child aged < 5 years or binary indication of severe acute malnutrition |
| Collected respiratory specimen | | Sample type indicated as spontaneous or induced sputum, nasopharyngeal aspirate, gastric aspirate, or other respiratory sample | Indication if two respiratory samples were collected and if not, how many were collected | Sample type indicated as expectorated sputum, gastric aspirate or nasopharyngeal aspirate | Indication of expectorated sputum collection and of nasopharyngeal aspirate collection (binary) |
| Xpert conducted | | Type of Xpert test (MTB-Rif or MTB Rif Ultra) | Type of Xpert test (MTB-Rif or MTB Rif Ultra) | Type of Xpert test (MTB-Rif or MTB Rif Ultra) | Type of Xpert test (MTB-Rif or MTB Rif Ultra) |

| | | | | | |
|---|---|---|---|---|---|
| Mtb final result | | Final Xpert result (positive or negative). This is the highest result as multiple samples were collected per child | Mtb complex detected or trace result | Mtb complex detected or trace result | Mtb complex detected or trace result |
| TB exposure < 12 months | | TB exposure at 24 months | Indication of TB exposure in the past 12 months | Indication of TB exposure in the past 12 months | Indication of TB exposure in the past year |
| **Variables for CXR evaluation** | | | | | |
| Cavities | | Presence of cavities at enrolment evaluated by central expert reviewer (binary) | Presence of cavities at enrolment evaluated by central expert reviewer (binary) | Presence of cavities at enrolment evaluated by central expert reviewer (binary) | Presence of cavities at enrolment evaluated by by central expert reviewer (binary) |
| Enlarged lymph nodes | | Presence of enlarged lymph nodes at enrolment evaluated by central expert reviewer (binary) | Presence of enlarged lymph nodes at enrolment evaluated by central expert reviewer (binary) | Presence of cavities at enrolment evaluated by central expert reviewer (binary) | Presence of enlarged lymph nodes at enrolment evaluated by central expert reviewer (binary) |
| Opacities | | Presence of opacities at enrolment evaluated by central expert reviewer (binary) | Presence of opacities at enrolment evaluated by central expert reviewer (binary) | Presence of interstitial opacities at enrolment evaluated by central expert reviewer (binary) | Presence of opacities at enrolment evaluated by central expert reviewer (binary) |
| Miliary pattern | | Presence of miliary patterns at enrolment | Presence of miliary patterns at enrolment | Presence of miliary at enrolment evaluated | Presence of miliary patterns at enrolment |

| | | | | | |
|---|---|---|---|---|---|
| | | evaluated by central expert reviewer (binary) | evaluated by central expert reviewer (binary) | by central expert reviewer (binary) | evaluated by central expert reviewer (binary) |
| Effusion | | Presence of effusion at enrolment evaluated by central expert reviewer (binary) | Presence of effusion at enrolment evaluated by central expert reviewer (binary) | Presence of pleural, pericardial or peritoneal effusion at enrolment evaluated by central expert reviewer (binary) | Presence of effusion at enrolment evaluated by central expert reviewer (binary) |
| Airway compression | | **Missing/not collected** | **Missing/not collected** | Presence of large airway compression at enrolment evaluated by central expert reviewer (binary) | Presence of large airway compression at enrolment evaluated by central expert reviewer (binary) |
| Diagnostic classification | | Based on NIH criteria (2015) | Based on NIH criteria (2015) | Based on NIH criteria (2015) | Based on NIH criteria (2015) |

Most variables were able to be used or minorly manipulated to fit the WHO definitions to be used in the TDA. There were two cases where changes had to be made; 1) as TB contact was recorded in variations including no time specification, within 12 months, or within 24 months, for this study we took any TB contact as positive. 2) Night sweats was entirely missing/not collected in the TB-Speed HIV cohort and we thus conducted simple imputation based on a logistic regression to address the missingness [1].

Abbreviations: SAM: severe acute malnutrition, NIH: National Institutes of Health, Mtb: mycobacterium tuberculosis, BMI: Body Mass Index, PCR: polymerase chain reaction, MUAC: mid upper arm circumference, WFA: weight-for-age, TDA: Treatment Decision Algorithm

## Supplemental Table 4. Outcome definitions used in study, based on NIH case definitions [5].

| | Categories | Definitions |
|---|---|---|
| TB classification | Unlikely | Bacteriological confirmation NOT obtained AND criteria for “unconfirmed tuberculosis” NOT met OR children that met “unconfirmed tuberculosis” but did not receive anti-tuberculosis treatment and were well at follow-up |
| | Unconfirmed | Bacteriological confirmation NOT obtained AND at least 2 of the following:<br>• Symptoms/signs suggestive of tuberculosis[1] (as defined)<br>• Chest radiograph[2] consistent with tuberculosis<br>• Close tuberculosis exposure or immunologic evidence of *Mtb* infection<br>• Positive response to tuberculosis treatment (requires documented positive clinical response on tuberculosis treatment - no time duration specified) |
| | Confirmed | Bacteriological confirmation obtained - requires *Mtb* to be confirmed (culture, Xpert MTB/RIF, or Xpert MTB/Rif Ultra assay) from at least 1 respiratory specimen |

[1]Symptoms/signs suggestive of TB include cough for more than 2 weeks, fever for more than 2 weeks, and poor weight gain or weight loss in the past 3 months. In young children (< 5 years), reduced playfulness or lethargy is also included.

[2]Chest X-rays were assessed by study-specific clinicians or external experts and defined as “attributable to TB”, “unlikely TB”, or “normal”. According to the operational handbook, common abnormalities in children include enlarged hilar and paratracheal lymph nodes, alveolar consolidation without visible cavities, miliary lesions, and pleural effusions [3].

Abbreviations: *Mtb, mycobacterium tuberculosis*. NIH, National Institute of Health.

medRxiv preprint doi: https://doi.org/10.64898/2026.07.07.26357441; this version posted July 20, 2026. The copyright holder for this preprint (which was not certified by peer review) is the author/funder, who has granted medRxiv a license to display the preprint in perpetuity.
It is made available under a CC-BY 4.0 International license .

## Supplemental Table 5. Score 1 development for Model 1, with bacteriological testing.

| Predictors | Level | OR | Model β coefficient | Final Predictor Score (x scaling factor: 38.2) |
|---|---|---|---|---|
| History of TB exposure | Yes | 0.7 | -0.3 | -13 |
| HIV status positive | Yes | 1.6 | 0.5 | 18 |
| Night sweats | Yes | 0.6 | -0.5 | -20 |
| Tachycardia | Yes | 1.6 | 0.5 | 19 |
| Tachypnoea | Yes | 1.9 | 0.7 | 26 |
| Bacteriological Testing | Positive (Xpert MTB/RIF ≥Medium or smear-positive) | 7.1 | 1.9 | 75 |
| Intercept | | - | -1.9 | -75.8 |
| Predicted probability cutoff, *P* | | - | 0.15 | 5.8 |
| Logit, *P* | | - | -1.723 | -65.8 |
| Severity diagnosis threshold (Logit [*P*] – intercept) | | - | 0.36 | 10.0 |

OR, odds ratio; *P*: Predicted probability cutoff; Xpert MTB/RIF ≥medium – medium, high, very high.

medRxiv preprint doi: https://doi.org/10.64898/2026.07.07.26357441; this version posted July 20, 2026. The copyright holder for this preprint (which was not certified by peer review) is the author/funder, who has granted medRxiv a license to display the preprint in perpetuity.
It is made available under a CC-BY 4.0 International license .

## Supplemental Table 6. Score 2 development for Model 2, without bacteriological testing.

| Predictors | Levels | OR | Model β coefficient | Final Predictor Score (× scaling factor: 79.4) |
|---|---|---|---|---|
| History of TB exposure | Yes | 1.2 | -0.4 | -29 |
| Night sweats | Yes | 0.7 | -0.4 | -28 |
| Tachycardia | Yes | 1.7 | 0.5 | 43 |
| Tachypnoea | Yes | 2.0 | 0.7 | 57 |
| Intercept | | - | -1.69 | -134.5 |
| Predicted probability cutoff, *P* | | - | 0.17 | 13.7 |
| Logit, *P* | | - | -1.568 | -124.5 |
| Severity diagnosis threshold (Logit [*P*] – intercept) | | - | 0.13 | 10.0 |

OR, odds ratio; *P*: Predicted probability cutoff;

medRxiv preprint doi: https://doi.org/10.64898/2026.07.07.26357441; this version posted July 20, 2026. The copyright holder for this preprint (which was not certified by peer review) is the author/funder, who has granted medRxiv a license to display the preprint in perpetuity.
It is made available under a CC-BY 4.0 International license .

Supplemental Table 7. Characteristics of children included in the individual patient dataset by parent study.

| Characteristic | RaPaed-TB N = 272 (43.0%) | UMOYA N = 163 (25.8%) | TB-Speed HIV N = 55 (8.7%) | TB-Speed Decentralisation N = 143 (22.6%) | p-value* |
|---|---|---|---|---|---|
| **Age (years) [median (IQR)]** | 3.1 (1.4, 6.1) | 1.7 (1.0, 3.3) | 4.5 (1.9, 6.3) | 3.4 (1.6, 5.9) | <0.001 |
| **Age (categories)** | | | | | <0.001 |
| 3 months to <2 years (%) | 102 (37.5%) | 96 (58.9%) | 14 (25.5%) | 37 (25.9%) | |
| 2 to <5 years (%) | 78 (28.7%) | 51 (31.3%) | 22 (40.0%) | 63 (44.1%) | |
| 5 to <10 years (%) | 92 (33.8%) | 16 (9.8%) | 19 (34.5%) | 43 (30.1%) | |
| **Sex** | | | | | 0.215 |
| Female (%) | 119 (43.8%) | 87 (53.4%) | 23 (41.8%) | 65 (45.5%) | |
| Male (%) | 153 (56.3%) | 76 (46.6%) | 32 (58.2%) | 78 (54.5%) | |
| **Weight/BMI for Age (Z score)** | | | | | 0.004 |
| > -1 (%) | 140 (51.5%) | 102 (62.6%) | 21 (38.2%) | 78 (54.5%) | |
| > -2 to < -1 (%) | 92 (33.8%) | 37 (22.7%) | 16 (29.1%) | 42 (29.4%) | |
| > -3 to < -2 (%) | 40 (14.7%) | 24 (14.7%) | 18 (32.7%) | 23 (16.1%) | |
| **CLHIV (%)** | | | | | <0.001 |
| Positive | 41 (15.1%) | 16 (9.8%) | 55 (100.0%) | 6 (4.2%) | |
| **NIH 2015 case definition [5]** | | | | | <0.001 |
| Confirmed tuberculosis (%) | 110 (40.4%) | 61 (37.4%) | 6 (10.9%) | 25 (17.5%) | |
| Unconfirmed tuberculosis (%) | 162 (59.6%) | 102 (62.6%) | 49 (89.1%) | 118 (82.5%) | |
| **Facility type** | | | | | <0.001 |
| Primary Healthcare (%) | 0 (0.0%) | 0 (0.0%) | 0 (0.0%) | 4 (2.8%) | |
| District Healthcare (%) | 0 (0.0%) | 0 (0.0%) | 0 (0.0%) | 139 (97.2%) | |
| Tertiary Healthcare (%) | 272 (100.0%) | 163 (100.0%) | 55 (100.0%) | 0 (0.0%) | |
| **Symptoms** | | | | | |
| Cough ≥ 2 weeks (%) | 192 (70.6%) | 74 (45.4%) | 43 (78.2%) | 118 (82.5%) | <0.001 |
| Fever ≥ 2 weeks (%) | 111 (40.8%) | 25 (15.3%) | 25 (45.5%) | 95 (66.4%) | <0.001 |
| Weight loss (%) | 160 (58.8%) | 88 (54.0%) | 24 (43.6%) | 86 (60.1%) | 0.139 |
| Night sweats (%) | 120 (44.1%) | 51 (31.3%) | 9 (16.4%) | 40 (28.0%) | <0.001 |
| Enlarged lymph nodes (%) | 52 (19.1%) | 15 (9.2%) | 16 (29.1%) | 27 (18.9%) | 0.003 |
| Tachycardia (%) | 32 (11.8%) | 6 (3.7%) | 8 (14.5%) | 7 (4.9%) | 0.003 |
| Tachypnoea (%) | 31 (11.4%) | 25 (15.3%) | 13 (23.6%) | 34 (23.8%) | 0.005 |

medRxiv preprint doi: https://doi.org/10.64898/2026.07.07.26357441; this version posted July 20, 2026. The copyright holder for this preprint (which was not certified by peer review) is the author/funder, who has granted medRxiv a license to display the preprint in perpetuity.
It is made available under a CC-BY 4.0 International license .

| | | | | | |
|---|---|---|---|---|---|
| **TB Exposure (%)** | 154 (56.6%) | 103 (63.2%) | 14 (25.5%) | 89 (62.2%) | <0.001 |
| **Xpert MTB/RIF or Ultra** | | | | | |
| *Mtb* Negative (%) | | | | | 0.018 |
| *Mtb* Positive, including trace (%) | 198 (72.8%) | 129 (79.1%) | 49 (89.1%) | 118 (82.5%) | |
| **Ziehl-Neelsen smear** | | | | | >0.999 |
| Negative (%) | 63 (94.0%) | 154 (94.5%) | 0 (0.0%) | 0 (0.0%) | |
| Positive (%) | 4 (6.0%) | 9 (5.5%) | 0 (0.0%) | 0 (0.0%) | |
| Not performed (%) | 205 | 0 | 55 | 143 | |
| **WHO eligibility criteria groups** | | | | | 0.408 |
| Xpert MTB/RIF or Ultra results: negative, trace, low, very low OR smear negative (%) | 260 (95.6%) | 150 (92.0%) | 53 (96.4%) | 135 (94.4%) | |
| Xpert MTB/RIF or Ultra results: Medium, high, very high OR smear positive (%) | 12 (4.4%) | 13 (8.0%) | 2 (3.6%) | 8 (5.6%) | |
| **Radiological Disease Severity** | | | | | <0.001 |
| Non-severe features (%) | 198 (72.8%) | 146 (89.6%) | 47 (85.5%) | 126 (88.1%) | |
| Severe features (%) | 74 (27.2%) | 17 (10.4%) | 8 (14.5%) | 17 (11.9%) | |

*Kruskal-Wallis rank sum test (Age as a continuous variable); Pearson's Chi-squared test

Supplemental Table 8. Diagnostic accuracy of the WHO-criteria where bacteriological testing available and in subgroups against radiological severity as the reference standard.

| | TP | FP | FN | TN | Sensitivity (95% CI)* | | Specificity (95% CI)* | |
|---|---|---|---|---|---|---|---|---|
| **Where CXR is unavailable and bacteriological testing is available** | | | | | | | | |
| WHO-criteria | 25 | 57 | 91 | 460 | 30.1% | 20.3 – 40.2 | 83.4% | 80.2 – 86.4 |
| Study | | | | | | | | |
| RaPaed-TB | 11 | 18 | 63 | 180 | 14.9% | 7.0–23.6 | 90.9% | 86.6–94.8 |
| UMOYA | 6 | 12 | 11 | 134 | 35.4% | 12.5–60.9 | 91.7% | 86.7–95.9 |
| TB-Speed Dec. | 6 | 19 | 11 | 107 | 34.5% | 12.5–57.1 | 85.0% | 78.5–91.0 |
| TB-Speed HIV | 2 | 8 | 6 | 39 | 24.5% | 0.0–60.0 | 83.0% | 71.2–93.6 |
| NIH 2015 diagnosis | | | | | | | | |
| Confirmed TB | 19 | 25 | 34 | 119 | 36% | 22.9–50 | 82.60% | 76.1–88.8 |
| Unconfirmed TB | 6 | 27 | 57 | 322 | 9.50% | 2.9–17.5 | 92.30% | 89.2–95.1 |
| Age | | | | | | | | |
| 3 months to <2 years | 13 | 28 | 38 | 170 | 25.5% | 13.2–37.5 | 85.8% | 80.7–90.9 |
| 2 to <5 years | 4 | 14 | 32 | 164 | 10.9% | 2.4–21.7 | 92.2% | 88.1–95.9 |
| 5 to <10 years | 8 | 15 | 21 | 126 | 27.2% | 10.7–44.0 | 89.3% | 84.0–94.5 |
| Weight for Age Z Score | | | | | | | | |
| > -1 | 11 | 19 | 47 | 264 | 19.0% | 9.1–29.3 | 93.2% | 90.1–95.9 |
| > -2 to < -1 | 5 | 16 | 29 | 137 | 14.4% | 3.2–26.9 | 89.4% | 84.4–94.0 |
| > -3 to < -2 | 9 | 22 | 15 | 59 | 37.0% | 17.9–56.3 | 72.9% | 63.1–82.1 |
| HIV status | | | | | | | | |
| Children living with HIV | 5 | 11 | 22 | 80 | 18.4% | 4.2–34.4 | 88.0% | 80.4–94.4 |
| Children without HIV | 20 | 46 | 69 | 380 | 22.3% | 14.0–31.8 | 89.2% | 86.2–92.0 |
| Healthcare Level | | | | | | | | |
| District Healthcare | 6 | 17 | 11 | 105 | 34.4% | 11.8–57.2 | 86.1% | 79.7–92.0 |

| | | | | | | | | |
|---|---|---|---|---|---|---|---|---|
| Tertiary Healthcare | 19 | 38 | 80 | 353 | 19.4% | 12.0–27.5 | 90.3% | 87.4–93.1 |
| TB contact History | | | | | | | | |
| History of TB exposure | 8 | 33 | 46 | 273 | 14.8% | 6.1–24.6 | 89.2% | 85.7–92.5 |
| No history of TB exposure | 17 | 24 | 45 | 187 | 27.3% | 16.7–39.0 | 88.6% | 84.2–92.5 |

*Mean sensitivity and specificity after bootstrapping 1000 times.

TB: Tuberculosis; TP: True Positives; TN: True Negatives; FP: False Positives; FN: False Negatives. NIH 2015 diagnosis: National Institute of Health (NIH 2015) clinical case definitions; CI: Confidence Intervals; WHO: World Health Organisation.

Supplemental Table 9. Diagnostic accuracy of the WHO-criteria where bacteriological testing unavailable and in subgroups against radiological severity as the reference standard.

| | TP | FP | FN | TN | Sensitivity (95% CI) | | Specificity (95% CI) | |
|---|---|---|---|---|---|---|---|---|
| **Where CXR is unavailable and bacteriological testing is unavailable** | | | | | | | | |
| WHO-criteria | 11 | 39 | 105 | 478 | 21.7% | 10.4 – 34.6 | 81.9% | 78.8 – 84.9 |
| Study | | | | | | | | |
| RaPaed-TB | 3 | 14 | 71 | 184 | 4.1% | 0.0–8.8 | 92.9% | 88.9–96.4 |
| UMOYA | 2 | 5 | 15 | 141 | 11.8% | 0.0–30.8 | 96.5% | 93.6–99.3 |
| TB-Speed Dec. | 4 | 13 | 13 | 113 | 22.9% | 5.0–42.9 | 89.8% | 84.3–94.6 |
| TB-Speed HIV | 2 | 7 | 6 | 40 | 24.5% | 0.0–60.0 | 85.1% | 73.5–95.2 |
| NIH 2015 diagnosis | | | | | | | | |
| Confirmed TB | 5 | 8 | 48 | 141 | 9.6% | 2.1–19.0 | 94.6% | 90.8–98.0 |
| Unconfirmed TB | 6 | 31 | 57 | 337 | 9.6% | 3.2–17.1 | 91.6% | 88.8–94.2 |
| Age | | | | | | | | |
| 3 months to <2 years | 7 | 18 | 44 | 180 | 13.9% | 4.3–24.5 | 90.8% | 86.8–94.8 |
| 2 to <5 years | 2 | 9 | 34 | 169 | 5.4% | 0.0–13.9 | 94.9% | 91.5–97.8 |
| 5 to <10 years | 2 | 12 | 27 | 129 | 6.9% | 0.0–17.9 | 91.4% | 86.5–95.9 |
| Weight for Age Z Score | | | | | | | | |
| > -1 | 2 | 6 | 56 | 277 | 3.4% | 0.0–8.9 | 97.9% | 96.0–99.3 |
| > -2 to < -1 | 1 | 13 | 33 | 140 | 2.8% | 0.0–9.1 | 91.4% | 87.0–95.4 |
| > -3 to < -2 | 8 | 20 | 16 | 61 | 33.1% | 15.8–52.4 | 75.4% | 65.9–84.4 |
| HIV status | | | | | | | | |
| Children living with HIV | 4 | 10 | 23 | 81 | 14.9% | 2.8–29.0 | 89.1% | 82.0–95.6 |
| Children without HIV | 7 | 29 | 82 | 397 | 7.7% | 2.5–13.6 | 93.2% | 90.7–95.6 |
| Healthcare Level | | | | | | | | |
| District Healthcare | 4 | 12 | 13 | 110 | 22.9% | 4.5–44.4 | 90.2% | 84.7–95.2 |
| Tertiary Healthcare | 7 | 26 | 92 | 365 | 7.1% | 2.7–12.5 | 93.3% | 90.8–95.6 |

| | | | | | | | | |
|---|---|---|---|---|---|---|---|---|
| TB contact History | | | | | | | | |
| History of TB exposure | 1 | 19 | 53 | 287 | 1.8% | 0.0–6.2 | 93.8% | 91.2–96.4 |
| No history of TB exposure | 10 | 20 | 52 | 191 | 16.0% | 7.0–26.4 | 90.5% | 86.3–94.2 |

*Mean sensitivity and specificity after bootstrapping 1000 times.

TB: Tuberculosis; TP: True Positives; TN: True Negatives; FP: False Positives; FN: False Negatives. NIH 2015 diagnosis: National Institute of Health (NIH 2015) clinical case definitions; CI: Confidence Intervals; WHO: World Health Organisation.

medRxiv preprint doi: https://doi.org/10.64898/2026.07.07.26357441; this version posted July 20, 2026. The copyright holder for this preprint (which was not certified by peer review) is the author/funder, who has granted medRxiv a license to display the preprint in perpetuity.
It is made available under a CC-BY 4.0 International license .

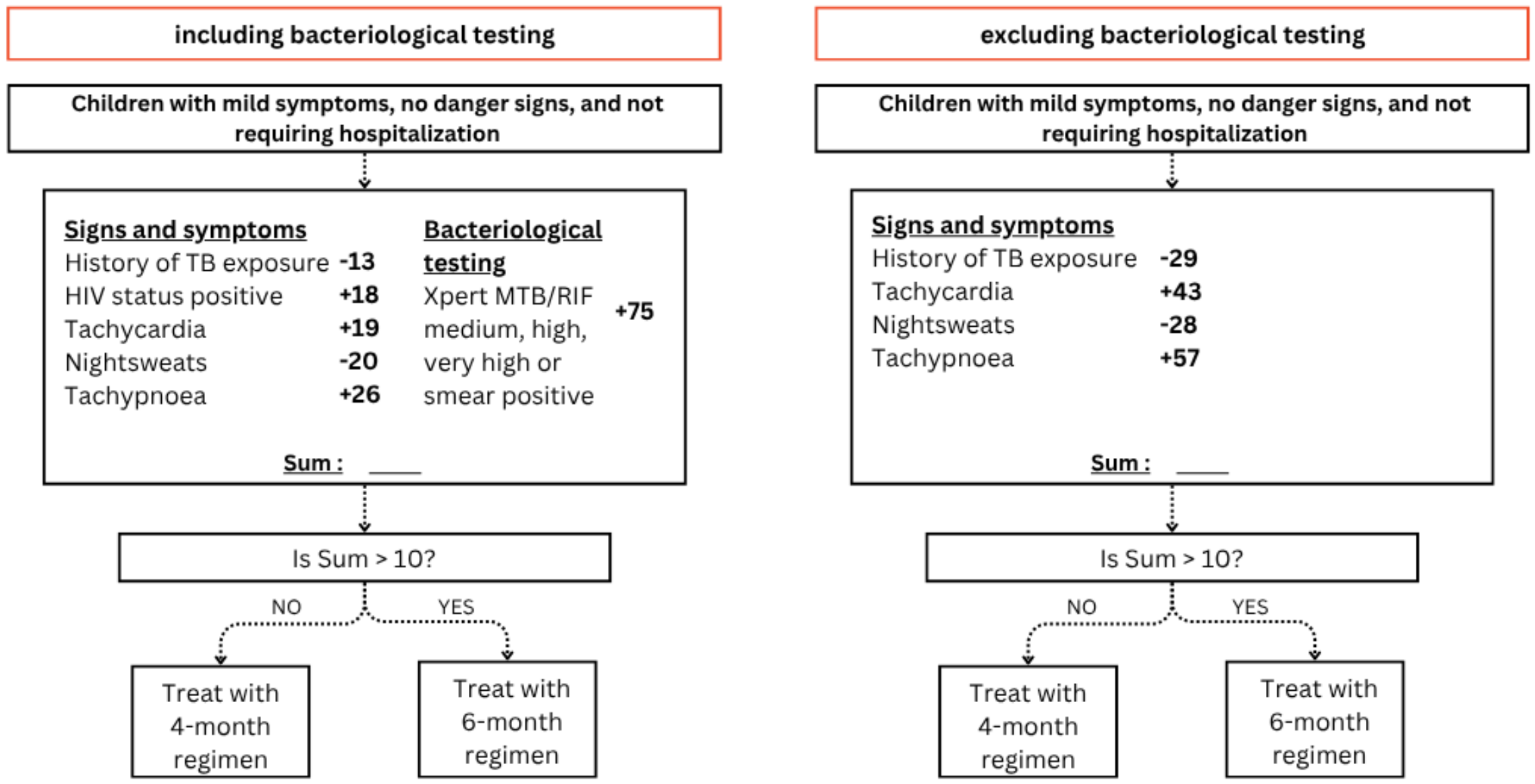


**Supplemental Figure 1. Scoring system schematic for possible use in practice for different settings alongside the WHO Treatment Decision Algorithms in the absence of chest X-ray.** Here score 1 is based on model 1 and refers to absence of chest X-ray, but with bacteriological testing. Score 2 is based on model 2 and refers to absence of chest X-ray and without bacteriological testing.

Supplemental Table 10. Diagnostic accuracy of the developed score with bacteriological testing for predicting CXR severity in different subgroups.

| | TP | FP | FN | TN | Sensitivity (95% CI)* | | Specificity (95% CI)* | |
|---|---|---|---|---|---|---|---|---|
| **Where CXR is unavailable and bacteriological testing is available** | | | | | | | | |
| Score 1 | 48 | 117 | 68 | 400 | 41.1% | 32.4-49.5 | 77.3% | 73.6-80.8 |
| Study | | | | | | | | |
| RaPaed-TB | 24 | 30 | 50 | 168 | 32.5% | 22.2–43.1 | 84.9% | 80.0–89.7 |
| UMOYA | 10 | 29 | 7 | 117 | 59.3% | 33.3–83.3 | 80.1% | 73.5–86.4 |
| TB-Speed Dec. | 9 | 25 | 8 | 101 | 53.1% | 31.2–77.8 | 80.0% | 73.5–86.7 |
| TB-Speed HIV | 5 | 33 | 3 | 14 | 63.0% | 25.0–100.0 | 30.1% | 17.9–44.4 |
| NIH 2015 diagnosis | | | | | | | | |
| Confirmed TB | 25 | 36 | 28 | 113 | 47.1% | 33.0–59.6 | 75.9% | 68.6–82.7 |
| Unconfirmed TB | 23 | 81 | 40 | 287 | 36.5% | 25.0–47.9 | 78.2% | 74.0–82.2 |
| Age | | | | | | | | |
| 3 months to <2 years | 23 | 49 | 28 | 149 | 45.7% | 31.8–59.6 | 75.2% | 69.3–80.6 |
| 2 to <5 years | 11 | 36 | 25 | 142 | 30.2% | 15.4–45.0 | 79.7% | 73.3–85.6 |
| 5 to <10 years | 14 | 32 | 15 | 109 | 47.3% | 28.8–66.2 | 77.2% | 71.0–83.7 |
| Weight for Age Z Score | | | | | | | | |
| > -1 | 24 | 50 | 34 | 233 | 41.8% | 28.9–54.5 | 82.2% | 77.9–86.6 |
| > -2 to < -1 | 12 | 39 | 22 | 114 | 35.1% | 20.0–51.7 | 74.2% | 67.3–80.8 |
| > -3 to < -2 | 12 | 28 | 12 | 53 | 50.6% | 28.8–70.8 | 65.2% | 54.7–74.7 |
| HIV status | | | | | | | | |
| Children living with HIV | 15 | 56 | 12 | 35 | 56.0% | 37.7–73.2 | 38.5% | 28.5–48.8 |
| Children without HIV | 33 | 61 | 56 | 365 | 37.3% | 27.8–47.5 | 85.7% | 82.2–88.9 |
| Healthcare Level | | | | | | | | |

| | | | | | | | | |
|---|---|---|---|---|---|---|---|---|
| District Healthcare | 9 | 23 | 8 | 99 | 53.4% | 29.4–76.9 | 81.0% | 74.2–87.7 |
| Tertiary Healthcare | 39 | 92 | 60 | 299 | 39.4% | 29.5–49.3 | 76.6% | 71.9–80.7 |
| TB contact History | | | | | | | | |
| History of TB exposure | 15 | 36 | 39 | 270 | 28.0% | 16.8–40.2 | 88.1% | 84.4–91.7 |
| No history of TB exposure | 33 | 81 | 29 | 130 | 53.3% | 41.6–66.1 | 61.7% | 55.9–68.6 |

*Mean sensitivity and specificity after bootstrapping 1000 times.

TB: Tuberculosis; TP: True Positives; TN: True Negatives; FP: False Positives; FN: False Negatives. NIH 2015 diagnosis: National Institute of Health (NIH 2015) clinical case definitions; CI: Confidence Intervals.

Supplemental Table 11. Diagnostic accuracy of the developed scores without bacteriological testing for predicting CXR severity in different subgroups.

| | TP | FP | FN | TN | Sensitivity (95% CI)* | | Specificity (95% CI)* | |
|---|---|---|---|---|---|---|---|---|
| **Where CXR is unavailable and bacteriological testing is unavailable** | | | | | | | | |
| Score 2 | 36 | 88 | 80 | 429 | 30.9% | 22.6-40.4 | 83.0% | 79.5-86.3 |
| Study | | | | | | | | |
| RaPaed-TB | 19 | 29 | 55 | 169 | 25.9% | 15.8–36.9 | 85.3% | 80.4–90.0 |
| UMOYA | 5 | 21 | 12 | 125 | 30.0% | 8.3–53.6 | 85.7% | 79.7–91.2 |
| TB-Speed Dec. | 8 | 24 | 9 | 102 | 47.3% | 25.0–71.8 | 80.8% | 73.5–87.7 |
| TB-Speed HIV | 4 | 14 | 4 | 33 | 49.9% | 15.4–85.2 | 70.4% | 56.0–82.7 |
| NIH 2015 diagnosis | | | | | | | | |
| Confirmed TB | 14 | 19 | 39 | 130 | 26.3% | 14.7–38.2 | 87.4% | 82.1–92.3 |
| Unconfirmed TB | 22 | 69 | 41 | 299 | 34.8% | 24.0–47.5 | 81.4% | 77.6–85.0 |
| Age | | | | | | | | |
| 3 months to <2 years | 18 | 39 | 33 | 159 | 35.4% | 21.8–50.0 | 80.2% | 74.3–85.6 |
| 2 to <5 years | 10 | 23 | 26 | 155 | 27.3% | 13.1–42.2 | 86.9% | 81.1–91.6 |
| 5 to <10 years | 8 | 26 | 21 | 115 | 27.3% | 12.0–45.3 | 81.8% | 75.4–88.1 |
| Weight for Age Z Score | | | | | | | | |
| > -1 | 15 | 37 | 43 | 246 | 25.8% | 15.7–38.1 | 86.9% | 82.9–90.7 |
| > -2 to < -1 | 11 | 29 | 23 | 124 | 31.7% | 16.4–48.3 | 80.7% | 74.5–86.5 |
| > -3 to < -2 | 10 | 22 | 14 | 59 | 42.3% | 22.6–61.9 | 72.7% | 63.0–81.4 |
| HIV status | | | | | | | | |
| Children living with HIV | 9 | 23 | 18 | 68 | 32.7% | 15.8–50.0 | 74.7% | 65.3–83.1 |
| Children without HIV | 27 | 65 | 62 | 361 | 30.5% | 21.5–39.8 | 84.7% | 81.5–87.9 |
| Healthcare Level | | | | | | | | |
| District Healthcare | 8 | 23 | 9 | 99 | 47.4% | 23.5–72.5 | 81.0% | 74.0–87.7 |
| Tertiary Healthcare | 28 | 64 | 71 | 327 | 28.3% | 20.0–38.3 | 83.7% | 79.8–87.4 |

| | | | | | | | | |
|---|---|---|---|---|---|---|---|---|
| TB contact History | | | | | | | | |
| History of TB exposure | 11 | 29 | 43 | 277 | 20.5% | 10.3–32.5 | 90.4% | 87.1–93.6 |
| No history of TB exposure | 25 | 59 | 37 | 152 | 40.6% | 28.6–54.2 | 72.2% | 65.4–77.6 |

*Mean sensitivity and specificity after bootstrapping 1000 times.

TB: Tuberculosis; TP: True Positives; TN: True Negatives; FP: False Positives; FN: False Negatives. NIH 2015 diagnosis: National Institute of Health (NIH 2015) clinical case definitions; CI: Confidence Intervals.

medRxiv preprint doi: https://doi.org/10.64898/2026.07.07.26357441; this version posted July 20, 2026. The copyright holder for this preprint (which was not certified by peer review) is the author/funder, who has granted medRxiv a license to display the preprint in perpetuity.
It is made available under a CC-BY 4.0 International license .

## References

1. Olbrich L, Larsson L, Dunbar R, Dodd PJ, Palmer M, Huyen Ton Nu Nguyet M, et al. Diagnostic accuracy of the WHO tuberculosis treatment decision algorithms for children with presumptive tuberculosis: An individual participant data meta-analysis. PLoS Med. 2025;22: e1004610-. Available: https://doi.org/10.1371/journal.pmed.1004610

2. Steyerberg EW. Statistics for Biology and Health Clinical Prediction Models A Practical Approach to Development, Validation, and Updating Second Edition. Available: http://www.springer.com/series/2848

3. WHO. Module 5: Management of tuberculosis in children and adolescents WHO operational handbook on tuberculosis. 2022.

4. Graham SM, Cuevas LE, Jean-Philippe P, Browning R, Casenghi M, Detjen AK, et al. Clinical Case Definitions for Classification of Intrathoracic Tuberculosis in Children: An Update. Clinical Infectious Diseases. 2015;61. doi:10.1093/cid/civ581
